# China’s universal two-child policy and depressive symptoms among women at childbearing age: a difference-in-difference analysis based on the China Family Panel Study

**DOI:** 10.1017/S2045796026100547

**Published:** 2026-03-26

**Authors:** Ruoxi Ding, Dianqi Yuan, Xiaohan Zhu, Yushan Du, Chao Guo

**Affiliations:** 1 Peking University Sixth Hospital, Peking University Institute of Mental Health, NHC Key Laboratory of Mental Health (Peking University), National Clinical Research Center for Mental Disorders (Peking University Sixth Hospital), Beijing, China; 2 Social Statistics Department, University of Manchester, Manchester, UK; 3 Max Planck Institute for Demographic Research (MPIDR), Rostock, Germany; 4 Institute of Population Research, Peking University, Beijing, China

**Keywords:** depressive symptoms, population high-quality development, population policy, universal two-child policy, women at childbearing age

## Abstract

**Aims:**

Limited studies have conducted a comprehensive investigation on the impact of China’s birth policy change on the mental health among women of childbearing age. This study aimed to explore the potential impact of China’s Universal two-child policy on depressive symptoms among women of childbearing age, based on national-representative, longitudinal survey data.

**Methods:**

Data we employed in this study were derived from the China Family Panel Study (CFPS) for the waves of 2012, 2014, 2016, 2018 and 2020. We included 7481 currently married females (17079 for pooled sample) aged 20–40 years. Depressive symptoms were assessed using the Kessler 6 Rating Scale (K6) and the Center for Epidemiologic Studies Depression Scale (CES-D). All scores were standardized for analysis. We employ the difference-in-difference model to investigate the association between the implementation of the Universal Two-child Policy (UTP) and women’s depressive symptoms.

**Results:**

Women in the exposed group, after implementing UTP, had a standardized score of depressive symptoms 0.10 higher (95% CI: 0.03–0.16, *p* = 0.007) than during the pre-intervention period after controlling for multiple covariates. They also faced a higher risk of having moderate or severe depressive symptoms (OR = 1.45, 95% CI: 1.12–1.87, *p* = 0.004). The stratified analysis revealed that the negative impact of UTP on mental health was pronounced among women with advanced age, low education, medium family income, only male offspring before UTP, and no new birth after UTP.

**Conclusion:**

We observed that the implementation of the UTP was associated with increased depressive symptoms among married women of childbearing age in China, with significant heterogeneity across different sociodemographic groups. Greater attention should be paid to the complex psychological conditions of women of childbearing age when adjusting fertility policies, which is crucial to prevent women from suffering poor mental health and to advance high-quality development in population health.

## Introduction

The childbearing age period (20–40 years old) is the lifespan of highest risk for developing mental health problems among women, regardless of whether they have given birth or not (Marcus and Heringhausen, 2009). Data from the National Survey on Drug Use and Health (NSDUH) suggested that in the United States, 1 out of 10 women of reproductive age experienced at least one major depressive episode during the past year (Farr *et al.*, 2012). As a vital dynamic and life-altering process that brings about ongoing transitions of identity, motherhood could present a permanent shift in a woman’s life and elevate the vulnerability to affect mood (Holton *et al.*, 2010). The associated biological and hormonal changes, the tedious housework and overwhelmed childcare with isolation and loss of social identity are all potentially undesirable and stressful life events following pregnancy and childbirth (Gameiro *et al.*, 2014). And for those without children, although the trajectory may be divergent, the motherhood- or childbearing-related issue that correlates to multiple aspects of life is also likely to have significant implications for women’s psychological well-being (Maximova and Quesnel-Vallée, 2009). Infertility and involuntary childlessness have been recognized as a multidimensional and long-lasting stressor to cause anxiety, distress and depression that require continuous emotional adjustments (Maximova and Quesnel-Vallée, 2009; Klemetti *et al.*, 2010).

Fertility in China has long been regulated by governmental policy. The One-child Policy (OCP) that was initiated in 1979 in response to the fears of overpopulation and the related social, economic and environmental challenges, has raged numerous debates over the positive impacts, including improvement in gender equality and a fall in maternal mortality, and negative consequences, such as the deprivation of reproductive choices, skewed sex ratio and accelerated population ageing (Hesketh and Zhu, 1997; Hesketh *et al.*, 2005). In November 2013, the Chinese government launched a Partial Two-child Policy incentivizing couples to have a second child if either parent was an only child. Subsequently, the Universal two-child policy was officially announced in October 2015, granting all families in China the right to have two children (Zeng and Hesketh, 2016). Even though this policy is inevitably speculative and may fall short of expectations, it is certain that it will substantially meet most of the couple’s reproductive preference, reduce the anxiety and stress of single-girl families with traditional preference for sons and successfully eliminate the adverse health outcomes related to economic punishment due to the so-called out-of-quota second pregnancies or child (Zeng and Hesketh, 2016).

Whereas existing research has primarily focused on the impacts of birth policy changes on women’s reproductive decisions, delivery methods, pregnancy complications and birth outcomes (Li *et al.*, 2019; Zhang *et al.*, 2020), few studies have specifically examined the psychological consequences of the transition from OCP to UTP oamong women of reproductive age. On the one hand, the introduction of the UTP may alleviate anxiety, stress and depressive symptoms caused by previously suppressed fertility desires. On the other hand, it may also impede progress in gender equality – which had previously liberated women from intense reproductive pressures – increase the risk of perinatal depressive symptoms resulting from multiple births, and incur discordance of reproductive intentions between couples, or even across different generations (Xiao *et al.*, 2019). Therefore, this study aimed to explore the potential impact of China’s UTP on the mental health of women of childbearing age, using a natural experimental design and drawing on the theoretical framework of social policy and gender health equity. Based on national-representative, longitudinal survey data with a robust method, this research seeks to elucidate how broad population policies differentially affect women’s psychosocial well-being. The findings will help anticipate the potential outcomes of current further adjustments, such as the Three-Child Policy, within China’s population policy, aiding people in better addressing reproductive-related health issues amidst China’s low fertility context.

## Methods

### Data source and study sample

**Figure 1.**
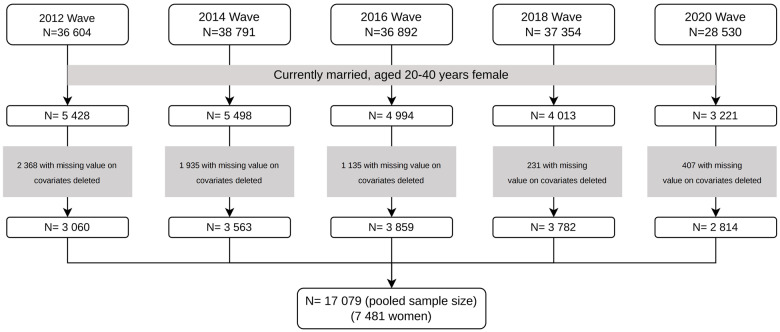
Flowchart of sample selection.

Data we employed in this study were derived from the China Family Panel Study (CFPS) for the waves of 2012, 2014, 2016, 2018 and 2020. CFPS is a nationally representative survey launched in 2010. This biennial follow-up survey covered 25 provinces, cities and autonomous regions in mainland China, aiming to collect information at individual, household and community levels, including a wide range of data on demographic and socioeconomic characteristics, health conditions and health service utilization. CFPS adopted a multi-stage stratified sampling method to reduce operation costs and implicit stratification to increase efficiency. At the first two stages in the sampling process, official administrative entities were used, and the final onsite sampling stage used frames constructed from field-drawn maps of dwelling units, not official household registers. The survey sampled all members of selected households, therefore allowing us to identify the information of the individual’s spouse, children and parents. The baseline survey was conducted in 2010 and covered 14 960 households, 33 600 adults and 8 990 children. The detailed information of the survey design has been reported elsewhere (Xie and Hu, 2014). Since China implemented the UTP in January 2016, we linked the 2012, 2014, 2016, 2018 and 2020 waves of CFPS data as a longitudinal dataset of an unbalanced panel to examine the impact of UTP. Since our study is focused on married females of childbearing age, we included 7481 currently married females (3 060, 3 563, 3 859, 3 782 and 2 814 women from 2012, 2014, 2016, 2018 and 2020 waves, and total 17 709 observations for the pooled sample) aged 20–40 years during the survey window from 2012 to 2020 with no missing value on socioeconomic information, CESD score and other health status (Fig. 1).

### Study design

We employ a quasi-experimental research design which is an effective approach for investigating the causal relationships between the implementation of the Universal two-child policy (UTP) and women’s depressive symptoms. The difference-in-difference (DID) approach is key to evaluating interventions to inform health policymakers and future strategies (Godard-Sebillotte *et al.*, 2019). This approach can effectively disentangle the impact of intervention from the permanent disparities between the intervention and control groups, as well as the temporal trends in the outcome that are unrelated to the intervention of UTP.

### Measures

We regard the survey year before 2016 (2012 and 2014) as the pre-UTP period and the survey year after 2016 (2016, 2018 and 2020) as the post-UTP period. Before the implementation of UTP, China implemented a selective two-child policy in 2014. The implementation of this policy allows couples to have two children as long as one of the couples is an only child. Therefore, UTP removed the restrictions on couples where neither spouse was from a one-child family, while it did not affect families where at least one of the spouses was an only child or families who had already had two or more children. We categorized women who were neither only children themselves nor had spouses who were only children, or who had either one or no children before 2016, as the UTP-exposed group. Women who were either only children themselves or whose spouses were only children, or who already had two or more children, were categorized as the control group. The impact of UTP is estimated by comparing the differences between two changes in outcomes: (i) changes in depressive symptoms between the pre- and post-UTP periods within the UTP-exposed group and (ii) the pre- and post-UTP periods in the control group.

Depressive symptoms were employed as the outcome variable. The questionnaire modules regarding depressive symptoms in the CFPS are different across waves. In 2010 and 2014, depressive symptoms were assessed based on the Kessler 6 Rating Scale (K6), which is a six-item scale asking how often the respondents experienced depression, nervousness, agitation, hopelessness, psychological fatigue, and worthlessness during the last month. The responses for the K6 items are on a 5-point scale: 0 (never), 1 (once a month), 2 (2–3 times a month), 3 (2–3 times a week), or 4 (almost every day). The total scores of these six items range from 0 to 24, with a higher score indicating more frequent or more severe depressive symptoms. In the 2012, 2016 and 2018 waves, items of depressive symptoms were adapted from the Center for Epidemiologic Studies Depression Scale (CES-D). The CES-D scale comprises 20 items, with 16 items measuring negative feelings and 4 items measuring positive emotions. The respondents are asked about the number of days on which they experienced depressive symptoms during the last 7 days. Responses are on a 4-point scale: 0 ‘rarely’ (less than 1 day), 1 ‘some days’ (1–2 days), 2 ‘occasionally’ (3–4 days), and 3 ‘most of the time’ (5–7 days). The positive questions were further reversed to calculate the final score, which ranges from 0 to 60, with higher values indicating more severe or more frequent depressive symptoms. To reduce respondent burden and refusal rate, the assessment switched to a shorter version of the scale – the CES-D scale to a subset of eight items (hereafter CES-D8) in 2016, 2018 and 2020.

It is noteworthy that both the K6 and CES-D8 scales have been widely validated and demonstrate good reliability and validity in general population studies. The K6 has shown high internal consistency (Cronbach’s *α* > 0.80) and strong discriminant validity in distinguishing clinical cases of depression, while the CES-D8 also exhibits acceptable reliability (*α* typically between 0.70 and 0.80) and correlates highly with the full CES-D scale. Therefore, to ensure continuity, we employed CES-D8 to assess the depressive symptoms in the 2012, 2016, 2018 and 2020 waves, and further standardized the total scores (CES-D8 and K6 in 2014) of each wave. The standardized score of depressive symptoms was the main outcome variable. Additionally, we used a binary outcome of depression for sensitivity analyses, which was categorized into ‘having moderate or severe depressive symptoms’ (5 or higher in K6 score, or 7 or higher in CES-D8, coded 1) or ‘mentally healthy’ (coded 0).

Control variables included the following: age (modelled as a continuous variable in years), residence (categorized as urban or rural based on official household registration status), education (categorized into three levels: primary school or below, junior high or high school, and junior college or above), financial status (operationalized as a categorical variable defined by tertiles of annual family income per capita: low, medium, or high), the gender composition of children, if any (categorized as: no child, only girl(s), both boy(s) and girl(s), or only boy(s)), self-rated health (‘In general, how would you rate your health?’) scored on a scale from 0 to 4 (where 0 = very poor and 4 = very good), and chronic conditions (defined as a binary variable indicating the presence or absence of any physician-diagnosed chronic disease within the past six months).

### Statistical analysis

We employed a linear regression model for the continuous outcome variable (standardized score of depressive symptoms to estimate the impact of UTP on mental health among married women of childbearing age. To integrate multiple waves of the longitudinal survey, we structured the data in a panel format and included survey wave fixed effects in all regression models to control for time-specific shocks and systematic differences across waves. We adjusted results for several potential confounders including age, residence, education, financial status, self-rated health score, chronic conditions and gender of children. The regression model was:

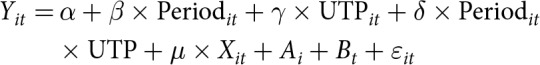



Here, 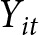 is the standardized score of depressive symptoms of an individual *i* in the year *t*. 
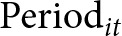
 is a variable indicating whether the observation was during the UTP implemented period. 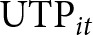 is a variable indicating whether the observation was in the UTP-exposed group or control group. 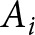 is the individual fixed effect, 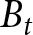 is a vector of dummy variables for a year-fixed effect, 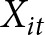 is a set of varying time, individual-level covariates and 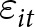
is the error term. The key parameter of interest is 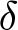, the DID estimate, which measures the pre-post change in depressive symptoms between the UTP-exposed group and control group, thereby indicating the impact of UTP on women’s depressive symptoms. We performed stratified analyses by education, financial status and gender of children with the same model mentioned above to examine how socioeconomic and cultural factors moderate the policy’s mental health effects. This approach is grounded in the rationale that these variables capture critical dimensions of vulnerability – including resource constraints and son preference-related family pressures – that may lead to differential impacts of fertility policies on psychological well-being.

The DID analysis was based on the ‘common trend assumption’, stating that the intervention and control group would have the same variation trend in the pre-intervention periods. Thus, a parallel trend analysis was employed to assess whether the impact measured can be attributed to the implementation of UTP in the sensitivity analyses. Moreover, we used the binary outcome (having moderate or severe depressive symptoms or not) with logistic regression to reanalyse and check the robustness of the results. Furthermore, considering the variation in depression measurement methods across some survey years, we conducted additional analysis using only the 2012, 2016 and 2018 surveys, all of which utilized the CES-D8 for depression measurement, to further examine the robustness of the results. Additionally, we accounted for potential within-individual correlation across waves by clustering standard errors at the individual level in all regression models. All analyses were performed using the STATA version 17.0 for Mac (Stata Corp, College Station, TX, USA).

## Results

### Sample characteristics

The descriptive statistics are presented in Table 1. The pooled sample included 17 079 observations in total, with 6 623 before and 10 456 after the implementation of UTP. The mean age was 31.89 (SD: 5.07), 51.50% lived in urban areas, and 42.97% had a junior high or high school degree. The family income per capita ranged from 0 to 4 100 000 CNY, with a mean of 21 915 CNY. The average self-rated health score was 2.26, and 1 263 (7.40%) have chronic conditions. About 9.3% of women have no children at survey time, and 34.52% have only male offspring. A total of 5 561 (32.56%) subjects were categorized as the UTP-exposed groups.

**Table 1.** Sample characteristics

Characteristics		Total sample (*N* = 17079) (Mean [SD]/*N* [%])	Pre-UTP^a^ (*N* = 6623) (Mean [SD]/*N* [%])	Post UTP^a^ (*N* = 10456) (Mean [SD]/*N* [%])
Age (years)		31.89 (5.07)	32.05 (5.42)	31.79 (4.84)
Residence	Urban	8796 (51.50)	3225 (48.69)	5571 (53.28)
	Rural	8283 (48.50)	3398 (51.31)	4885 (46.72)
Education	Primary school or below	4379 (25.64)	2559(38.64)	1820 (17.41)
	Junior high or high school	7339 (42.97)	3252 (49.10)	4087 (39.09)
	Junior college or above	5361 (31.39)	812 (12.26)	4549 (43.51)
Financial status	Low	5466 (32.00)	2153 (32.51)	3313 (31.69)
	Medium	6327 (37.05)	2398 (36.21)	3929 (37.58)
	High	5286 (30.95)	2072 (31.28)	3214 (30.74)
Self-rated health score		2.26(1.07)	2.22 (1.11)	2.28 (1.04)
Chronic conditions	No	15816 (92.60)	6093 (92.00)	9723 (92.99)
	Yes	1263 (7.40)	530 (8.00)	733 (7.01)
Number of children	0	1588 (9.30)	556 (8.39)	1032 (9.87)
	1	7813 (45.75)	3295 (49.75)	4518 (43.21)
	2+	7678 (44.95)	2772 (41.85)	4906 (46.92)
Gender of children before UTP	No child	2764 (16.18)	564 (8.52)	2200 (21.04)
	Only girl(s)	4298 (25.17)	1836 (27.59)	2462 (23.55)
	Boy & girl	4122 (24.13)	1783 (26.92)	3339 (22.37)
	Only boy(s)	5895 (34.52)	2440 (36.84)	3455 (33.04)
UTP exposure	No	11518 (67.44)	4038 (60.97)	7480 (71.54)
	Yes	5561 (32.56)	2585 (39.03)	2976 (28.46)
Standardized score of depressive symptoms		0.56 (0.93)	0.44 (0.99)	0.64 (0.89)
Moderate or severe depressive symptoms	No	11801 (69.10)	5006 (75.59)	6795 (64.99)
	Yes	5278 (30.90)	1617 (24.41)	3661 (35.01)

a UTP, Universal two-child policy; *N* = observations.

### Depressive symptoms

**Figure 2.**
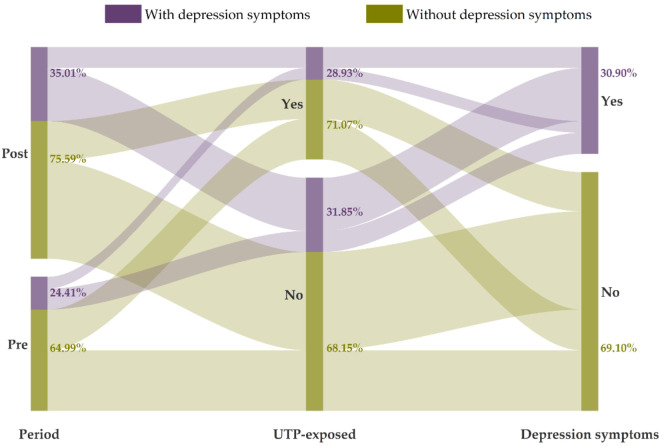
Alluvial diagram of the depression symptoms changes by the UTP exposed group and time.

*Note*: UTP, Universal two-child policy. Purple numbers represent the proportion of depression across different groups, while olive denotes the proportion without depression.

The mean standardized score of depressive symptoms was 0.56, with 0.44 for pre-UTP period and 0.64 for post-UTP period, and the mean score for UTP-unexposed and UTP-exposed group were 0.58 and 0.52. About 30.90% have moderate or severe depressive symptoms, with 24.41% for pre-UTP period and 35.01% for post-UTP period, and the proportion for UTP-unexposed and UTP-exposed group were 31.85% and 28.93% (Fig. 2). Depressive symptoms were significantly more common in post UTP period in comparison to that in pre-UTP period, whereas its difference between UTP-unexposed and UTP-exposed group was not pronounced. Thus, further analysis on the impact of UTP is required to exclude the time trend effects.

### Difference in difference estimates

**Figure 3.**
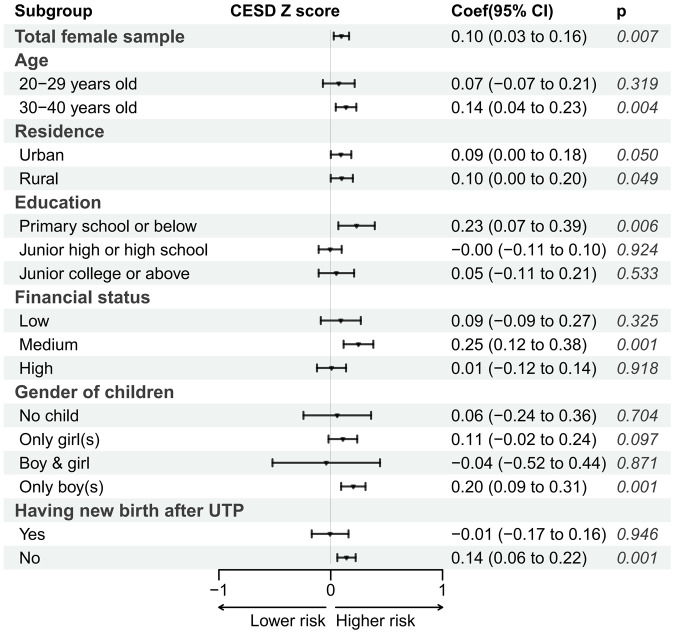
The impact of UTP on the standardized score of depressive symptoms.

*Note*: UTP, Universal two-child policy. Covariates including sociodemographic characteristics and physical health status were controlled.

Figure 3 describes the estimate of the DID model, which determines the impact of UTP on depressive symptoms of married women of childbearing age. After adjusting for sociodemographic characteristics and physical health status, the coefficient of UTP is 0.10 (*P* = 0.007). That is to say, the standardized score of depressive symptoms of women in the UTP-exposed group after implementing UTP was 0.10 higher than the pre-intervention period, which indicated that UTP significantly increased the risk of developing depressive symptoms among married women of childbearing age. The estimates of crude model and adjusted model were provided in Appendix table 1.

Figure 3 also presents the stratified analysis of the DID models. The age-group-specific result showed that the coefficient of UTP is 0.07(*P* = 0.319) and 0.14 (*P* = 0.004) for the group of 20–29, and 30–40 years old, suggesting that the impact of UTP was only significant among women in older age. The impact was also significant in both urban and rural subgroups. Education-specific subgroup result indicated that the impact of UTP was only significant among women with low educational background (0.23, *P* = 0.006). And similar pattern was also observed for subgroup analysis by gender of financial status, children before UTP and new birth after UTP, with the impact only pronounced among women with medium family income per capita (0.25, *P* = 0.001), women with only male off-spring before UTP (0.20, *P* = 0.001), and women have no new birth after UTP (0.14, *P* = 0.001).

### Sensitivity analysis

**Figure 4.**
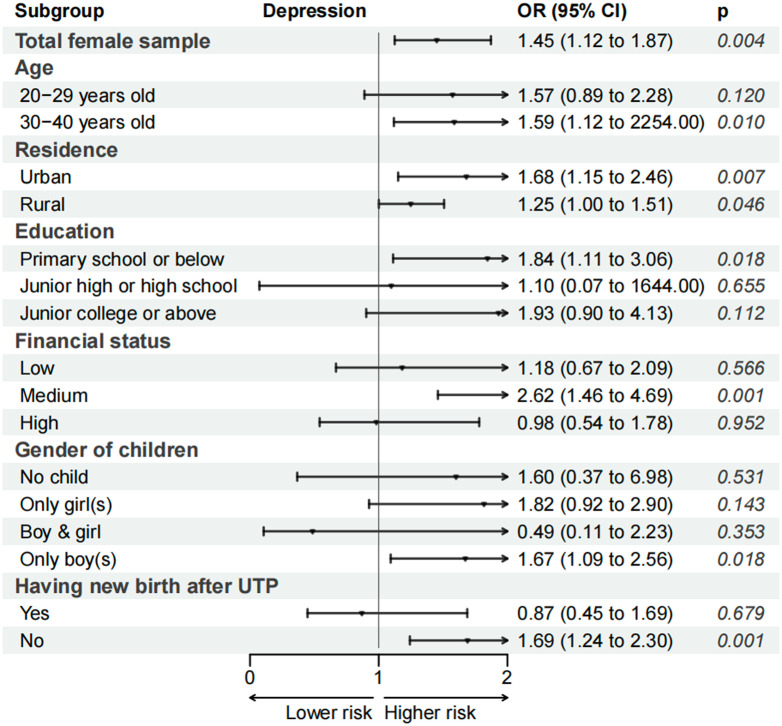
The impact of the Universal two-child policy on the risk of having moderate or severe depressive symptoms (subgroup analyses).

Appendix Fig. 1 shows the parallel trend test on the impact of UTP. As shown, the pre-intervention period estimates were not statistically significant, and the estimates became insignificant one year after the implementation of UTP, then were significant in the next year (2018), but finally dropped 3 years after the policy implementation. Overall, there were no differences in the pre-intervention trends for depressive symptoms among married women. Figure 4 shows the estimate of the DID model based on binary outcome variables using logistic regressions. Overall, the implementation of UTP increased the odds of developing moderate or severe depressive symptoms among women in the UTP-exposed group (OR:1.45, *P* = 0.004). Additionally, the pattern of stratified analyses was similar to the result based on standardized scores of depressive symptoms, with the impact of UTP being significant among women from both urban and rural side, in groups of 30–40 years old, junior high or high school, with medium financial status, with only male offspring before UTP and have no new birth after UTP. Additionally, in the analysis using only the 2012, 2016 and 2018 surveys, all of which employed the CES-D8 for depression measurement, we still observed similar results (refer to Appendix Table 2). Therefore, the sensitivity analysis supports our inference that the impact of UTP on women’s depressive symptoms is not caused by additional factors but is generated by the implementation of this new birth policy.

## Discussion

Utilizing nationwide survey data from China between 2012 and 2020, this study examined the mental health effects of the transition from the OCP to the Universal Two-Child Policy among women of reproductive age. Our analysis revealed that the introduction of the UTP was associated with a significant increase in depressive symptoms among the policy-targeted population, with an estimated 45% elevation in the risk of moderate-to-severe depression. Additionally, the stratified analysis revealed heterogeneity across different sociodemographic groups, with the negative effect on mental health pronounced among women with advanced age, low education, medium family income, only male offspring before UTP, and no new birth after UTP. These findings contribute to the literature pool with evidence regarding the broader health impacts, especially mental health, of population policy shifts beyond the reproductive process.

First of all, UTP was found to have a negative impact on women’s mental health, which aligns with existing literature suggesting that rapid shifts in fertility policies can exacerbate psychological distress among vulnerable subgroups (He *et al.*, 2025). The sharp increase in birth to multiparous women could be one of the potential explanations. Research on the change in births during the enactment of this policy showed that there were an additional 5.4 million births to multipara during the first 18 months after the implementation of UTP (Li *et al.*, 2019). It has been suggested that mothers with multiple births had 40% greater risks of developing moderate or severe, 9-month postpartum, depressive symptoms, compared with mothers of singletons (Choi *et al.*, 2009). Besides, an increased number of children could significantly impact on mother’s career and well-formed lifestyle, as well as the financial strains, which could all be potential stressors triggering anxiety and depressive symptoms.

Another possible reason involves the discordance of birth preference within the family (Gove and Geerken, 1977; Lu *et al.*, 2020). The implementation of the UTP may directly add challenge to women’s low-fertility intention, which has been protected by the enforcement of the OCP during the last 30 years. This finding is supported by previous work highlighting how familial pressure and relational conflicts act as significant predictors of perinatal depression (Wu *et al.*, 2025). A survey and qualitative study on pregnancy intention among Chinese women under the new birth policy showed that some women with no preference on second child would be compromised to their husbands or parents (Xiang and Yang, 2015; Ningning and Yue, 2018). Therefore, either compromising to husband or parents’ desire to involuntarily bearing the second child, or insisting on the original birth intention to risk damaging marital and family relationships could act as adverse life events and significantly increase women’s psychological stress. Additionally, the stratified analysis by age groups showed that the impact of UTP was particularly significant among there among women of advanced age. In contrast, no statistically significant effect was observed among women with younger age, which may be attributed to their greater flexibility in family planning, lower physiological risks associated with pregnancy, and relatively higher career development flexibility during this life stage. The failure of conception or giving birth to a second child among women with high fertility intention may serve as another pathway linking to negative mental health consequence.

Second, stratified analyses indicated that the negative impact of UTP on mental health was pronounced among women with low educational background or medium financial status. Many researchers argued that gender discrimination and women’s empowerment were one of the potential pathway that leads to adverse mental health consequences (Stepanikova *et al.*, 2020). One study on Iranian women found a significant association between women’s education and their empowerment in reproductive health decision-making (Kiani *et al.*, 2016). Education is recognized as a pivotal factor for empowering women, which not only enhances individual skills but also equips individuals with the capacity to effectively communicate with the outside world and utilize resources to achieve domestic objectives, which include not only birth intentions, but also the arrangement of childcare and household tasks (Kabeer, 2005). The heterogeneity across different financial statuses was supported by an inverted J-shape relationship between fertility and income level, which concluded from the long-run historical data, showing that the fertility rate was highest at low-income level, declined as income rise, but recovered at high-income levels (Nakagaki, 2018). Consequently, it is rational to infer that women with medium family income may suffer more psychological stress from their elder generation for the discordance between the subjective low fertility intention and the traditional conception of ‘more children, more blessing’ after the relaxation of the original OCP. Besides, compared to low-income group who can rely on ‘investment in child-rearing’ or ‘raise children to provide against old age’ and high-income group who could give birth without consideration of the cost of raising, the middle-income group faces the relatively highest input–output ratio in child-rearing. Therefore, even if this group has high fertility intention, the conflict of fertility decision due to insufficient resources or support may affect their mental health status (Chen and Yip, 2017; Zhu and Hong, 2022).

Third, we also noticed that women with only boys were affected by the UTP, instead of women with only girls or no child. Son preference is a common cultural and social tradition rooted in Chinese Confucian thoughts, as well as in other Eastern Asia societies (Qian and Jin, 2018). One study from Korea indicated that son-favouring parents are more likely to avoid having a second child if the first one is a son (Kim and Lee, 2020). And previous findings also suggested that boys always require more frequent and effortful supervisory of parents than girls to ensure safety (Phelan *et al.*, 2014). One study based on the China Fertility Survey indicated that the attitudes of Chinese women towards having sons have changed from ‘a hope for having many sons’ to ‘the fear of having two sons’ (Shi and Yang, 2021). However, the desire for a second grandchild from the previous generation who always held the conception of ‘having a son and daughter is equal to happiness’ and ‘more children, more blessing’, which was discordant with the women’s intention, may contribute to negative mental health consequences. On the contrary, for those with only girls, the implementation of the UTP provides an opportunity to achieve their son-favouring fertility intention and thus improve the marital and family relationship, and thus lead to ameliorated stress and anxiety. Together with the findings of another stratified analysis, which showing that the negative impact of new birth policy was only significant among women who have no new birth after UTP, these results further denote that the low intention or the incapability to have the second child that conflicting with the relaxation of OCP were the potential mechanism that linked to women’s depressive symptoms.

### Implications

The negative impact of UTP on mental health among childbearing female may provide important policy implications. From the demographic perspective, the improvement in fertility rates in China has been far from satisfactory since the implementation of UTP, and even after the following Three-child policy. This may be attributed to the low fertility intention and conflicts between the fertility desire and the reality of inadequate social and economic support, which are also the potential factors contribute to adverse mental health outcomes, as suggested by our findings. It further underscore the importance of simultaneous support including childcare services, parenting allowance, etc. for population policy adjustment since the mental health may consequently affecting the reproductive attitudes and behaviours among childbearing women. From the perspective of population health, considering the substantial burden of depression, and the vital impact of reproductive behaviour on mental health among female population, it is essential to provide universal screening with early intervention and support, empowerment in fertility decisions, as well as comprehensive efforts to promote gender equality are vital to help women strike a balance between the desire for parenthood and personal or professional development, and thus alleviate the increasing burden of mental disorders.

### Limitation

Our analyses were subject to several limitations. First, the CFPS data employed self-reported information rather than a medical diagnosis to assess depressive symptoms, and other adjusted variables depend on single-item responses, which may have been affected by an individual’s recall bias and unwillingness to avoid embarrassment. Second, due to the absence of information in some waves of the CFPS adult survey, we failed to include fertility intention, marital relationship, etc. in our analyses. Future qualitative studies are needed to explore the underlying mechanism linking birth policy change to poor mental health. Third, the heterogeneity in measures of depressive symptoms across waves may also lead to potential bias, which could be minus since the sensitivity analysis using waves with the same scale (CES-D8) also supported our main findings. Despite these limitations, this study possesses notable strengths, including its use of a large-scale, nationally representative longitudinal dataset and its novel focus on the mental health effects of the Universal Two-Child Policy among married reproductive-age women in China. To our knowledge, it is the first to provide empirical evidence on this significant yet understudied topic over an extended period.

## Conclusion

We observed that the implementation of the Universal Two-child Policy was associated with increased depressive symptoms among married women of childbearing age in China, and those with advanced age, low education, medium financial status, only male offspring and no new birth after UTP were particularly vulnerable to this effect. Due to the ongoing efforts in China to adjust and optimize the population composition and promote high-quality development in population, universal screening and reasonable measures including women empowerment on fertility decisions, comprehensive actions on gender equality promotion, as well as sufficient and subsidized infant and childcare provision are crucial to prevent women from suffering poor mental health. Future studies should further explore the mechanisms behind these effects through mixed-method approaches. Moreover, longitudinal evaluations of subsequent pronatalist policies are recommended to assess their mental health implications.

## Supporting information

10.1017/S2045796026100547.sm001 Ding et al. supplementary material Ding et al. supplementary material

## Data Availability

The datasets that support this article are publicly available from the project of the CFPS. Questionnaires are free download at website (http://www.isss.pku.edu.cn/cfps/en/documentation/questionnaires/index.htm?CSRFT=YPAC-8N0M-L215-I0D4-BP4X-GPXD-IPHR-KM6Y) and the datasets can be obtained after sending a data user agreement to the CFPS team (http://www.isss.pku.edu.cn/cfps/download/index#/fileTreeList).

## Supplementary material

The supplementary material for this article can be found at https://doi.org/10.1017/S2045796026100547.

## References

[ref1] Chen M and Yip PS (2017) The discrepancy between ideal and actual parity in Hong Kong: fertility desire, intention, and behavior. *Population Research and Policy Review* 36, 583–605.

[ref2] Choi Y, Bishai D and Minkovitz CS (2009) Multiple births are a risk factor for postpartum maternal depressive symptoms. *Pediatrics* 123(4), 1147–1154. 19336374 10.1542/peds.2008-1619

[ref4] Gameiro S, van den Belt-dusebout AW, Bleiker E, Braat D, van Leeuwen FE and Verhaak CM (2014) Do children make you happier? sustained child-wish and mental health in women 11–17 years after fertility treatment. *Human Reproduction* 29(10), 2238–2246. 25205751 10.1093/humrep/deu178

[ref5] Godard-Sebillotte C, Karunananthan S and Vedel I (2019) Difference-in-differences analysis and the propensity score to estimate the impact of non-randomized primary care interventions. *Family Practice* 36(2), 247–251. 30768124 10.1093/fampra/cmz003 PMC6425463

[ref6] Gove WR and Geerken MR (1977) The effect of children and employment on the mental health of married men and women. *Social Forces* 56(1), 66–76.

[ref7] He Y, Wahab NETA and Muhamad H (2025) Fertility anxiety among Chinese women in the context of fertility policy relaxation: a systematic literature review. *International Journal of Social Welfare* 34(3), e70028.

[ref8] Hesketh T, Lu L and Xing ZW (2005) The effect of China’s one-child family policy after 25 years. *New England Journal of Medicine* 353(11), 1171–1176. 16162890 10.1056/NEJMhpr051833

[ref9] Hesketh T and Zhu W (1997) Health in China: The one child family policy: the good, the bad, and the ugly. *British Medical Journal* 314, 1685–1687. 9193296 10.1136/bmj.314.7095.1685 PMC2126838

[ref10] Holton S, Fisher J and Rowe H (2010) Motherhood: is it good for women’s mental health? *Journal of Reproductive and Infant Psychology* 28(3), 223–239.

[ref11] Kabeer N (2005) Gender equality and women’s empowerment: a critical analysis of the third millennium development goal 1. *Gender & Development* 13(1), 13–24.

[ref12] Kiani Z, Simbar M, Dolatian M and Zayeri F (2016) Correlation between social determinants of health and women’s empowerment in reproductive decision-making among Iranian women. *Global Journal of Health Science* 8(9), 312–321. 10.5539/gjhs.v8n9p312 PMC5064082 27157184

[ref13] Kim S and Lee S-H (2020) Son preference and fertility decisions: evidence from spatiotemporal Variation in Korea. *Demography* 57(3), 927–951. 32430894 10.1007/s13524-020-00875-7

[ref3] Ko JY, Farr SL, Dietz PM and Robbins CL (2012) Depression and treatment among U.S. pregnant and nonpregnant women of reproductive age, 2005-2009. *Journal of Womens Health* 21(8), 830–836. 10.1089/jwh.2011.3466 PMC4416220 22691031

[ref14] Klemetti R, Raitanen J, Sihvo S, Saarni S and Koponen P (2010) Infertility, mental disorders and well‐being–a nationwide survey. *Acta Obstetricia et Gynecologica Scandinavica* 89(5), 677–682. 20196679 10.3109/00016341003623746

[ref15] Li H-T, Xue M, Hellerstein S, Cai Y, Gao Y, Zhang Y, Qiao J, Blustein J and Liu J-M (2019) Association of China’s universal two child policy with changes in births and birth related health factors: national, descriptive comparative study. *British Medical Journal* 366, l4680. 31434652 10.1136/bmj.l4680 PMC6699592

[ref16] Lu L, Duan Z, Wang Y, Wilson A, Yang Y, Zhu L, Guo Y, Lv Y, Yang X and Yu R (2020) Mental health outcomes among Chinese prenatal and postpartum women after the implementation of universal two-child policy. *Journal of Affective Disorders* 264, 187–192. 32056749 10.1016/j.jad.2019.12.011

[ref17] Marcus SM and Heringhausen JE (2009) Depression in childbearing women: when depression complicates pregnancy. *Primary Care: Clinics in Office Practice* 36(1), 151–165. 19231607 10.1016/j.pop.2008.10.011 PMC2680254

[ref18] Maximova K and Quesnel-Vallée A (2009) Mental health consequences of unintended childlessness and unplanned births: gender differences and life course dynamics. *Social Science & Medicine* 68(5), 850–857. 19097676 10.1016/j.socscimed.2008.11.012 PMC3762744

[ref19] Nakagaki Y (2018) Fertility, female labor participation and income in East Asia. *International Journal of Development Issues* 17(1), 69–86.

[ref20] Ningning D and Yue Y (2018) Value orientation analysis of the desire to give a second birth under the backdrop of the universal two-child policy. *JiangHan Academic* 37(2), 41–48.

[ref21] Phelan KJ, Morrongiello BA, Khoury JC, Xu Y, Liddy S and Lanphear B (2014) Maternal supervision of children during their first 3 years of life: the influence of maternal depression and child gender. *Journal of Pediatric Psychology* 39(3), 349–357. 24357732 10.1093/jpepsy/jst090 PMC3959264

[ref22] Qian Y and Jin Y (2018) Women’s fertility autonomy in urban China: the role of couple dynamics under the universal two-child policy. *Chinese Sociological Review* 50(3), 275–309.

[ref23] Shi R and Yang H (2021) The fear of having two sons: a noteworthy preference for the gender of children. *China Population and Development Studies* 5, 138–152.

[ref24] Stepanikova I, Acharya S, Abdalla S, Baker E, Klanova J and Darmstadt GL (2020) Gender discrimination and depressive symptoms among child-bearing women: ELSPAC-CZ cohort study. *EClinicalMedicine* 20, 100297. 32300743 10.1016/j.eclinm.2020.100297 PMC7152827

[ref25] Wu S, Zhang Y, Wang H, Dai J, Ding W, Zhu X and Xu X (2025) Prepregnancy stressful life events and perinatal mental health disorders from pregnancy to 3 years after childbirth: an observational study. *American Journal of Preventive Medicine* 69(5), 108039. 40784466 10.1016/j.amepre.2025.108039

[ref26] Xiang H and Yang J (2015) The development trend of China’s population and population policy adjustment. *Western Forum* 25 (4), 40–48.

[ref27] Xiao M, Fu B, Mi C, Pan C, Zhu S and Lei J (2019) The pregnancy intention to have a second child and antenatal depressive symptoms in Chinese women under the new two-child-per-couple policy. Preprint. Available at: Research Square. 10.21203/rs.2.15650/v1.

[ref28] Xie Y and Hu J (2014) An introduction to the China family panel studies (CFPS). *Chinese Sociological Review* 47(1), 3–29.

[ref29] Zeng Y and Hesketh T (2016) The effects of China’s universal two-child policy. *The Lancet* 388(10054), 1930–1938. 10.1016/S0140-6736(16)31405-2 PMC5944611 27751400

[ref30] Zhang X, Chen L, Wang X, Wang X, Jia M, Ni S, He W and Zhu S (2020) Changes in maternal age and prevalence of congenital anomalies during the enactment of China’s universal two-child policy (2013–2017) in Zhejiang Province, China: an observational study. *PLoS Medicine* 17(2), e1003047. 32092053 10.1371/journal.pmed.1003047 PMC7039412

[ref31] Zhu W and Hong X (2022) Are Chinese parents willing to have a second child? Investigation on the ideal and realistic fertility willingness of different income family. *Early Education and Development* 33(3), 375–390.

