# Supplementary material for: China’s universal two-child policy and depressive symptoms among women at childbearing age: a difference-in-difference analysis based on the China Family Panel Study
Source: Epidemiol Psychiatr Sci. 2026 Mar 26;35:e18. doi: 10.1017/S2045796026100547 (PMC13122544; doi:10.1017/S2045796026100547)
Supplement: Ding et al. supplementary material [file S2045796026100547sup001.docx]

***Supplementary materials***

**China’s Universal Two-child Policy and depressive symptoms among women at childbearing age: A difference-in-differences analysis based on the China Family Panel Study**

Appendix Table 1. The impact of the Universal two-child policy on the standardized score of depressive symptoms (N=17709)

|  |  | **Model 1** | | **Model 2** | |
| --- | --- | --- | --- | --- | --- |
|  |  | Coefficient | 95%CI | Coefficient | 95%CI |
| **Universal two-child policy** | | 0.068^*^ | [0.002,0.135] | 0.095^**^ | [0.027,0.164] |
| **Age** |  | - | - | 0.005 | [-0.068,0.077] |
| **Residence** | Urban |  |  | 0.088^*^ | [0.012,0.164] |
|  | Rural | - | - | Ref | Ref |
| **Education** | Primary school or below |  |  | Ref | Ref |
|  | Junior high or high school | - | - | -0.018 | [-0.081,0.045] |
|  | Junior college or above |  |  | -0.012 | [-0.105,0.0797] |
| **Financial status** | Low | - | - |  |  |
|  | Medium |  |  | 0.004 | [-0.047,0.038] |
|  | High | - | - | -0.051 | [-0.105,0.005] |
| **Self-rated health score** |  | - | - | -0.117^***^ | [-0.136,-0.100] |
| **Chronic conditions** | No | - | - | Ref | Ref |
|  | Yes | - | - | 0.156^***^ | [0.093,0.220] |
| **Gender of children before UTP^1^** | No child | - | - | Ref | Ref |
|  | Only girl(s) | - | - | 0.043 | [-0.102,0.189] |
|  | Boy & girl | - | - | 0.059 | [-0.103,0.221] |
|  | Only boy(s) | - | - | -0.018 | [-0.159,0.124] |

^*^P<0.05,^**^P<0.01,^***^P<0.001;

^1^Universal two-child policy.


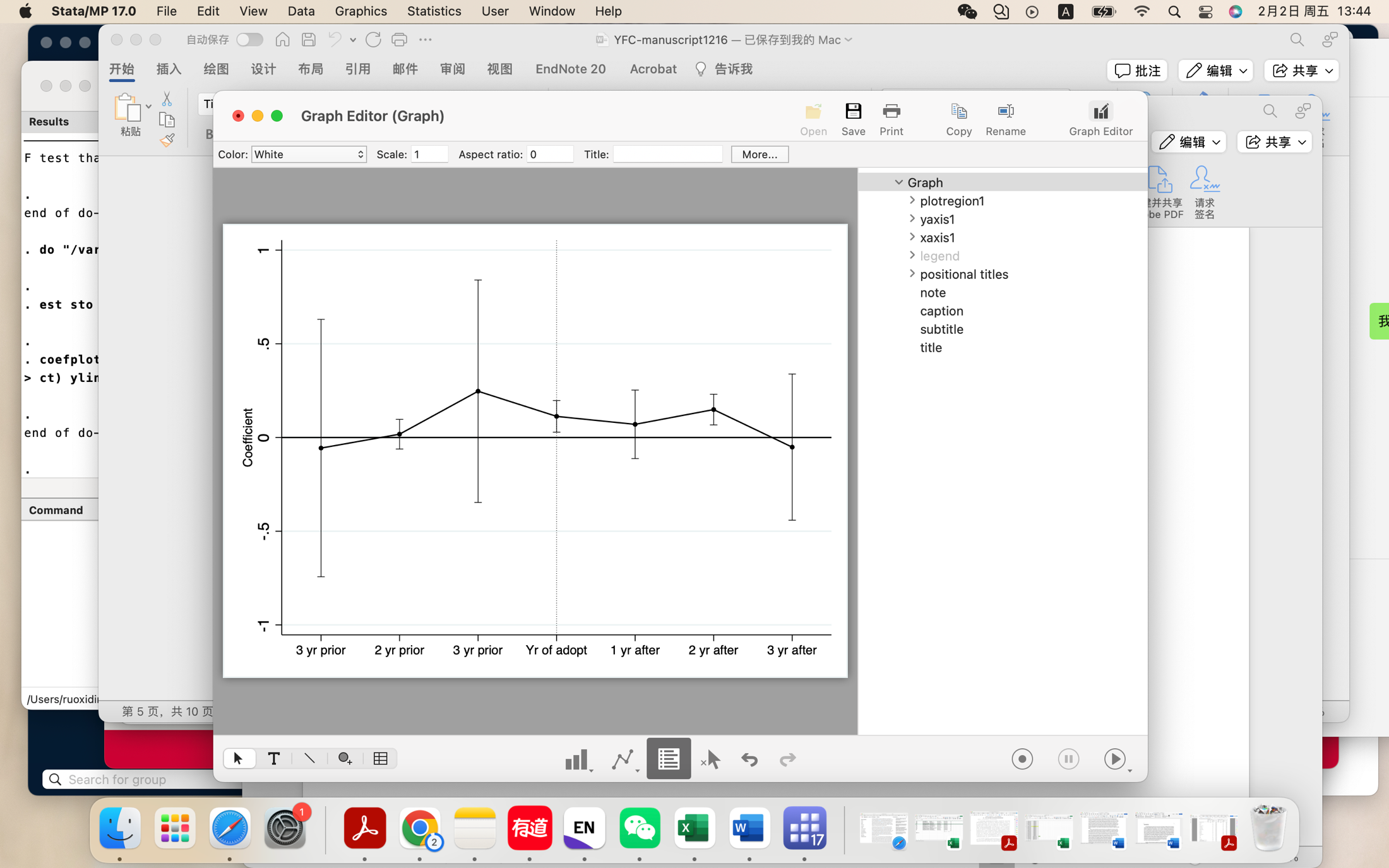


Appendix Figure 1. Parallel trend test on the impact of Universal two-child policy

Appendix Table 2. The impact of the Universal two-child policy on the standardized score of depressive symptoms (Robust analysis)

|  | Score of depressive symtoms (CES-D8, 2012, 2016 & 2018) | | | |
| --- | --- | --- | --- | --- |
|  | DID coefficient | 95% CI Upper | 95% CI Lower | P value |
| **Total female sample** | 0.615 | 0.252 | 0.979 | 0.001 |
| **By age group** |  |  |  |  |
| 20-29 years old | -0.134 | -1.072 | 0.803 | 0.779 |
| 30-40 years old | 0.832 | 0.359 | 1.306 | 0.001 |
| **By residence** |  |  |  |  |
| Urban | 0.351 | -0.153 | 0.855 | 0.172 |
| Rural | 0.061 | 0.034 | 1.18 | 0.038 |
| **By education** |  |  |  |  |
| Primary school or below | 1.105 | 0.264 | 1.947 | 0.01 |
| Junior high or high school | -0.012 | -0.592 | 0.568 | 0.967 |
| Junior college or above | 0.586 | -0.296 | 1.468 | 0.193 |
| **By financial status** |  |  |  |  |
| Low | 0.816 | -0.191 | 1.822 | 0.112 |
| Medium | 1.282 | 0.594 | 2.06 | 0.001 |
| High | -0.154 | -0.901 | 0.592 | 0.685 |
| **By gender of children** |  |  |  |  |
| No child | -2.206 | -4.669 | 0.256 | 0.079 |
| Only girl(s) | 0.183 | -0.504 | 0.869 | 0.602 |
| Boy & girl | -0.126 | -2.964 | 2.711 | 0.930 |
| Only boy(s) | 0.984 | 0.388 | 1.581 | 0.001 |
| **By having new birth after UTP^1^** |  |  |  |  |
| Yes | 0.749 | 0.326 | 1.173 | 0.001 |
| No | 0.833 | -0.279 | 1.945 | 0.142 |

^1^Universal two-child policy.
